# Electrocatalytic functional group conversion-based carbon resource upgrading

**DOI:** 10.1039/d4sc00175c

**Published:** 2024-04-02

**Authors:** Di Si, Xue Teng, Bingyan Xiong, Lisong Chen, Jianlin Shi

**Affiliations:** a Shanghai Key Laboratory of Green Chemistry and Chemical Processes, State Key Laboratory of Petroleum Molecular and Process Engineering, School of Chemistry and Molecular Engineering, East China Normal University Shanghai 200062 China lschen@chem.ecnu.edu.cn; b Shanghai Tenth People's Hospital, Shanghai Frontiers Science Center of Nanocatalytic Medicine, School of Medicine, Tongji University Shanghai 200072 P. R. China; c Institute of Eco-Chongming Shanghai 202162 China; d Shanghai Institute of Ceramics, Chinese Academy of Sciences Shanghai 200050 P. R. China

## Abstract

The conversions of carbon resources, such as alcohols, aldehydes/ketones, and ethers, have been being one of the hottest topics most recently for the goal of carbon neutralization. The emerging electrocatalytic upgrading has been regarded as a promising strategy aiming to convert carbon resources into value-added chemicals. Although exciting progress has been made and reviewed recently in this area by mostly focusing on the explorations of valuable anodic oxidation or cathodic reduction reactions individually, however, the reaction rules of these reactions are still missing, and how to purposely find or rationally design novel but efficient reactions in batches is still challenging. The properties and transformations of key functional groups in substrate molecules play critically important roles in carbon resources conversion reactions, which have been paid more attention to and may offer hidden keys to achieve the above goal. In this review, the properties of functional groups are addressed and discussed in detail, and the reported electrocatalytic upgrading reactions are summarized in four categories based on the types of functional groups of carbon resources. Possible reaction pathways closely related to functional groups will be summarized from the aspects of activation, cleavage and formation of chemical bonds. The current challenges and future opportunities of electrocatalytic upgrading of carbon resources are discussed at the end of this review.

## Abstract 2

The reported electrocatalytic upgrading reactions of carbon resources are summarized in four categories based on the types of functional groups from the aspects of activation, cleavage and formation of chemical bonds.

## Graphical abstract

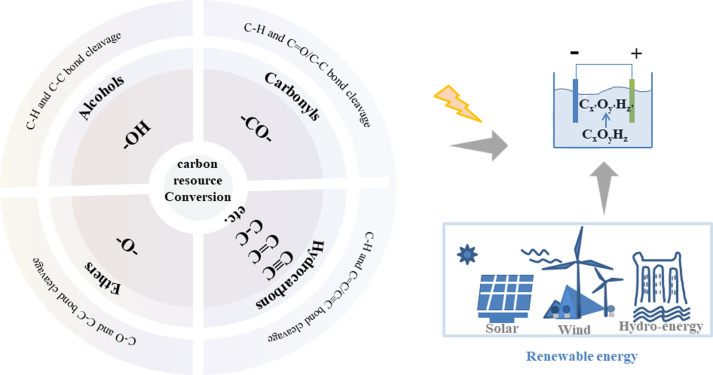

## 1. Introduction

The production of fuels, plastics, and pharmaceuticals on a large scale is greatly reliant on the capacity of the chemical industry. The chemical industry is inseparable from thermal catalytic processes, which play a fundamental role in converting various feedstocks into a diverse array of commodity chemicals. However, it heavily relies on finite fossil fuels as feedstocks and energy inputs, making it the largest energy consumer among all industrial sectors. To meet the growing demand for clean energy and address the environmental issues caused by industrial expansion, carbon peaking and carbon neutrality have gained significant attention worldwide. These topics have emerged as some of the most pressing concerns in current chemical and energy research.^1–4^ Consequently, the exploration of new methods for fuel and chemical synthesis is of utmost importance, with electrocatalysis being deemed as a highly potential avenue among them.^5^

Electrocatalysis is a green chemistry process that employs renewable and plentiful feedstocks such as air, water, and carbonaceous compounds, as well as renewable energy sources including wind, hydro, and solar power. This approach offers several advantages such as high activity and selectivity, 100% atom utilization efficiency, mild reaction conditions, and flexible modular production, making it a promising avenue for sustainable chemical synthesis.^6^ When powered by electricity, the electrocatalytic system can oxidize and reduce precursors at the anode and cathode respectively, without the need for chemical oxidants/reductants that may cause serious pollution. It has been confirmed that the electrocatalytic semi-hydrogenation conversion of acetylene to ethylene process is completely feasible through the technical and economic analysis.^7^ The well-controlled electric field acts as a strong nucleophilic/electrophilic agent, enabling high-precision activation of chemical bonds in the precursors for chemical reactions. The theoretically infinite redox range endows electrochemistry with the capability of oxidizing/reducing some of the most resilient compounds.

The common kinds of organic compounds include oxygen-containing organic compounds and hydrocarbons, which are classified according to their functional group. The functional groups commonly refer to atoms or clusters of atoms, including –C=C/C≡C, –OH, –CHO, –COOH, –NO_2_, –SO_3_H, –NH_2_ and RCO–, which belong to unsaturated hydrocarbons, alcohols or phenols, aldehydes, carboxylic acids, nitro compounds or nitriles, sulfonic acid organics, amines, and amides, respectively. The distinct properties exhibited by these compounds arise from the varying functional groups they possess. Furthermore, functional groups serve as reactive centers in organic chemical reactions. The essence of electrocatalytic conversion of organic matter lies in the transformation of these functional groups at the catalyst–electrolyte interface, involving sequential multistep electron transfer reactions that encompass the cleavage and formation of chemical bonds. Hence, the electrocatalytic conversion process is intricately intertwined with the functional group of the reactant, the applied potential and the electrocatalyst.^8,9^ Among these, electrocatalysts have garnered significant attention and are widely studied.^10–13^ Strategies to enhance their electrocatalytic performance are rapidly expanding, such as doping, atomic dispersion, nanostructures, and heterointerface engineering, all of which show effective utilization of the intrinsic atoms involved. It makes a significant contribution to the activity and selectivity of electrocatalysts.

**Fig. 1:**
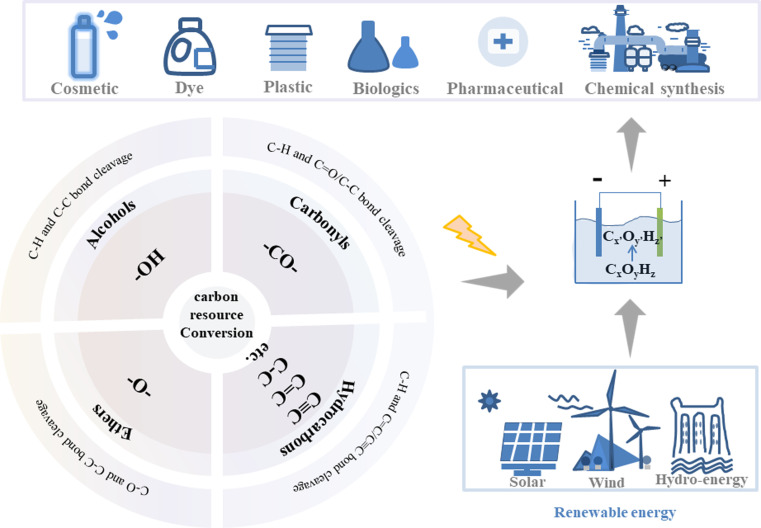
The illustration of electrocatalytic upgrading of carbon resources based on the classification of functional groups to generate commodity chemicals.

Although exciting progress has been achieved and even some reviews have been reported in this area recently, especially with a focus on exploration of valuable anodic oxidation or cathodic reduction reactions one by one, however, the reaction rules of these reactions are still missing and how to systematically find or design novel reactions in batches is still challenging. The properties and transformations of functional groups, which are critically important for carbon resource conversion reactions, may provide an approach to achieve the above goal.^14^ In this review, the properties of functional groups are described and the reported electrocatalytic upgrading reactions are summarized in four categories based on the classification of functional groups of carbon resources (Fig. 1): (i) electrocatalytic upgrading of alcohols, (ii) electrocatalytic upgrading of carbonyls, (iii) electrocatalytic upgrading of ethers, and (iv) electrocatalytic upgrading of hydrocarbons. Possible reaction paths for one functional group will be summarized from the aspects of activation, cleavage, and formation of chemical bonds. The current challenges and related prospects will be discussed, with the aim of benefiting the progress of electrocatalytic upgrading of carbon resources.

## 2. Electrocatalytic upgrading of alcohols

**Fig. 2:**
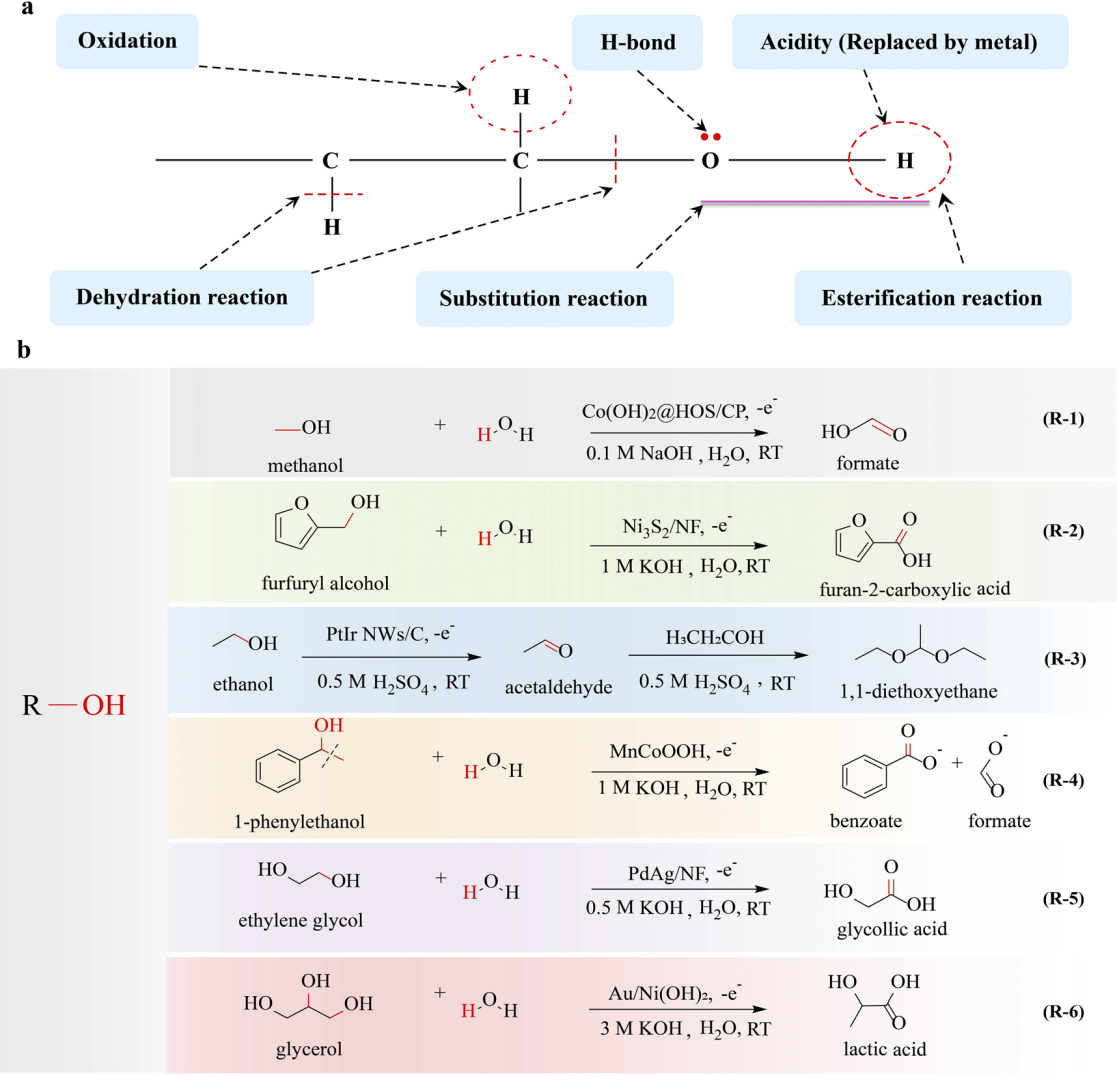
Electrocatalytic upgrading of alcohols. (a) The illustration of chemical properties of alcohols, and (b) the reports on alcohol electrocatalytic upgrade.

The hydroxyl group is the pivotal functional group of alcohols, playing a crucial role in various electrocatalytic conversion reactions and the properties of the hydroxyl group are crucial in determining the conversion efficiency of alcohols. The presence of a lone pair of electrons on the oxygen atom in the hydroxyl group enables it to attack positively charged atoms, which allows alcohols to undergo nucleophilic substitution reactions. Additionally, the electron-withdrawing induction effect of the hydroxyl group enhances the reactivity of the β-H in alcohols, favoring β-H elimination reactions. Moreover, alcohols are susceptible to oxidation reactions when hydrogen is present on their α-C (Fig. 2a). The alcohol oxidation reaction has attracted significant attention due to its potential applications in direct alcohol fuel cells, the conversion of biomass derivatives, and fine-chemical synthesis. Recently, electrocatalytic upgrading of various alcohols has been reported for synthesizing value-added products.^15–17^ In an electrocatalytic system, the electrooxidation of alcohol typically occurs in an aqueous electrolyte, and thus high purity hydrogen can also be obtained at the cathode, which is a hot topic. According to the number of hydroxyl groups contained in the molecule, alcohols can be divided into monohydric alcohols, diols and polyols.

### 2.1. Electrocatalytic upgrading of monohydric alcohol

Many studies have been reported on the production of high-value chemicals by electrocatalytic upgrading of monohydric alcohol focusing on the C–H breakage and nucleophilic substitution at the α-C.

#### 2.1.1. Transformation of the C–H bond

##### 2.1.1.1 Monohydric alcohols with one-carbon atoms

Methanol is not only a significant renewable and clean fuel, but also serves as a fundamental organic raw material for the production of diverse organic products, including formate, methylamine, and dimethyl sulfate. Fu *et al.*^18^ converted methanol to formate by eletrooxidation (MOR: CH_3_OH + 4OH^−^ → HCOOH + H_2_O + 4e^−^) (R-1 in Fig. 2). Benefiting from the efficient electrocatalyst Co(OH)_2_@HOS/CP, the elimination of α-H resulted in C–H bond breakage, followed by OH^−^ attack on the α-C, forming a C=O bond and formate was obtained. Sulfurization can regulate the electronic structure of active sites, optimizing the adsorption energy of OH^−^ and intermediates, which facilitates electron transfer from the antibonding orbital of metal–S bonds to metal–O bonds, thereby promoting C=O bond formation. In electrocatalysis, CO adsorption often acts as a poison or intermediate that causes poisoning, while the adsorption behavior of CO can be modulated by the rational design of electrocatalyst. Koper's group^19^ has concluded that CO adsorbed on an Au(111) surface promotes the electrocatalytic oxidation of methanol *via* facilitation of α-H elimination. It has been confirmed that the adsorbed CO alters the electrostatic surface potential, thereby enhancing the adsorption of OH^−^ on the hexagonal gold surface. This induces an enhancement in the electrostatic bonding component of OH^−^, which serves as the active species triggering C–H bond cleavage and C=O bond formation. Through co-catalysis, complex chemicals can be obtained. The conversion of methanol and ammonia to formamide was reported by Zhang's group.^20^ The electrochemical reaction achieved a formamide selectivity of 74.26% and a faradaic efficiency of 40.39%, in which the C–H bond in methanol is broken, leading to the formation of an aldehyde-like intermediate through methanol electrooxidation. Subsequently, this intermediate undergoes a nucleophilic attack by NH_3_, resulting in the formation of the C–N bond (cleavage of the C–H bond and O–H band).

##### 2.1.1.2 Monohydric alcohols with multi-carbon atoms

Inspired by the successful conversions of methanol, the upgrading of monohydric alcohols with more than one carbon atom has also been investigated. The electrocatalytic conversion of ethanol, isopropanol, *n*-propanol, benzyl alcohol, phenylethyl alcohol, and furfuryl alcohol to acetate, ethyl acetate, acetone, propionaldehyde, propionic acid, benzaldehyde, benzoic acid, phenylacetic acid, and 2-furoic acid has been reported, which is also focused on the cleavage of the C–H bond.^21–25^ Vizza^21^ has reported the electrooxidation of ethanol to acetate, in which the C–H bond is broken and a C=O bond is formed. A net profit analysis shows that electrocatalytic production of sodium acetate through ethanol oxidation is highly rewarding. The electrocatalytic conversion of cyclic polycarbonate alcohols into polycarbonate carboxylates is a crucial method, where the hydroxyl functional group is oxidized to carboxyl groups during the reaction process. You *et al.*^26^ prepared Ni_3_S_2_/NF as an electrocatalyst at the anode for the oxidation of biomass derived organic chemicals (R-2 in Fig. 2). The electrocatalytic oxidation of furfural alcohol and benzyl alcohol can effectively produce 2-furoic acid and benzoic acid with high efficiency and selectivity. With hydrogen on the α-C of these organic chemicals, it is easy for C–H bond cleavage and C=O and C–O bond formation. In addition to being converted into carboxylates, alcohols also serve as important precursors for the synthesis of aldehydes and ketones.^27^

Under uncontrolled oxidizing conditions, alcohols undergo oxidation to produce aldehydes, which are widely used in the chemical, wood, and textile industries.^28^ Conventional methods for oxidizing alcohols to aldehydes rely on the use of environmentally unfriendly stoichiometric oxidants such as chromium, manganese, and ruthenium salts. Electro-oxidation offers a sustainable and efficient alternative to traditional thermocatalytic methods. Due to the fact that aldehydes are susceptible to over-oxidation to acid, the selective oxidation of alcohols to corresponding aldehydes remains a significant challenge although numerous electrocatalysts have been developed.^29–31^ Currently, alkaline electrolyte is the most commonly utilized medium for the oxidation of alcohols; however, aldehydes are susceptible to condensation reactions and over-oxidation into carboxylate at high pH. It is considered that the neutral/near-neutral electrolyte would overcome these issues and enable selective oxidation of alcohols to aldehydes. It has been demonstrated^17^ that the electrooxidation of 5-hydroxymethyl furfural to 2,5-furandicarboxaldehyde in a neutral medium is more favorable than that in a alkaline medium with Ru_1_–NiO as the electrocatalyst, in which the C–H bond is broken to form a C=O bond. Wang's group^32^ realized the selective electrooxidation of alcohol to aldehyde on NiO by adjusting the local microenvironment and effectively separating the generated aldehyde from the reaction system. The conversion of 5-hydroxymethyl furfural to 2,5-furandicarboxaldehyde is achieved with high selectivity, which originates from the inhibition of aldehyde hydration by a salting-out effect, which specifically breaks the C–H bond.

Efficient cascade reactions are critical for the production of carbon-chain growth chemicals. Guo and coworkers^33^ reported the use of PtIr nanowires as catalysts for electrolytic production of multicarbon-1,1-dimethoxyethane (DEE) from the oxidation of ethanol *via* α-H elimination, in which electrocatalytic reaction was integrated with a condensation reaction driven by acid-catalysis (R-3 in Fig. 2). First, ethanol was dehydrogenated (C–H bond cleavage) and converted to acetaldehyde. Then, the acetaldehyde was transformed into DEE through acid-catalyzed acetalation (C–O–C bond formation), in which the shared electron pair between carbon and oxygen in the C–O bond was biased towards oxygen, enabling the nucleophilic substitution reaction to occur on the α-C of acetaldehyde, resulting in acetylation. The PtIr NWs/C catalyst shows excellent activity, achieving a high Faraday efficiency of 85% for DEE. Compared with other reactions where the carbon-chain is reduced or unchanged, the carbon-chain growth reaction is more difficult, and the reported literature is very rare.

#### 2.1.2. Transformation of the C–C bond

##### 2.1.2.1 Monohydric alcohols with multi-carbon atoms

In the electrocatalytic conversion of polycarbon monoalcohol, not only C–H bonds are broken but also C–C bonds can be cleaved to form new chemical bonds. Duan and coworkers^34^ synthesized an Mn-doped CoOOH electrocatalyst that effectively upgrades 1-phenylethanol into benzoate and formate through the cleavage of C(OH)–C bonds by electrocatalytic oxidation (R-4 in Fig. 2). During the reaction, 1-phenylethanol reacts with OH* species on the surface of MnCoOOH featuring the cleavage of C–H and C–C bonds and the formation of a C=O bond. It is noteworthy that an oxidative C–C cleavage process takes place to generate benzoate and formate as the final products. This method has the potential to be generalized to a range of carboxylates containing C(OH)–C or C(O)–C motifs, enabling the production of carboxylic acids with high yield. The cleavage of the C–C bond is possible due to the activity of the hydrogen in the hydroxyl functional group and the activity of the β-H. Duan and coworkers^35^ also reported an efficient electrocatalytic strategy for cleavage of the C_α_–C_β_ bond of β-O-4 linkage in 2-phenoxy-1-phenylethanol, exhibiting high yields for the products of aromatic aldehydes and phenols. The oxidative cleavage of the C(OH)–C bond to produce carboxylates is of significance for the upgrading of carbon resources.

### 2.2. Electrocatalytic upgrading of diols

In addition to monohydric alcohols, some exciting progress has been achieved in the electrocatalytic conversion of diols in recent years. Value added chemicals such as formic acid, acetic acid, 3-hydroxypropionic acid, and malonic acid have been obtained from the electrocatalytic conversion of ethylene glycol, 1,3-propanediol, 1,2-propanediol, 1,4-butanediol and 1,5-pentanediol with high efficiencies.^16,36–41^

#### 2.2.1. Transformation of the C–C bond

Ethylene glycol, the most representative diol with active α-H, is an important raw chemical that can be obtained from recycled plastics. Electrocatalysis offers a sustainable approach for the production of high-value chemicals by the oxidation of ethylene glycol. The cleavage of C–H and C–C bonds is prone to occur in diols, with the latter bond considered particularly crucial. Duan and coworkers^42^ developed a nickel-modified cobalt phosphide (CoNi_0.25_P) electrocatalyst for ethylene glycol oxidation, which achieved high Faraday efficiency of formate (>80%). Detailed characterization studies have revealed that the *in situ* formed low-crystalline metal oxy(hydroxide) serves as the active site for ethylene glycol oxidation, potentially explaining the high selectivity towards the targeted formate product.

#### 2.2.2. Transformation of the C–H bond

The production of C_2+_ organic chemicals without undergoing C–C cleavage during the electrocatalytic process is considered more attractive due to significantly enhanced product values. Noble metal-based catalysts exhibit high selectivity towards C–C bond retention products in the electrooxidation of alcohols.^11^ In a typical reaction, noble metal surfaces facilitate the electrooxidation of H_2_O or OH^−^ at low potentials, generating reactive oxygen species, which are commonly found in the form of adsorbed hydroxyl (OH*) groups. The OH* attacks alcohol, leading to C–H cleavage without causing C–C bond breakage; therefore value-added chemicals such as aldehyde, ketone, and carboxylic acid can be obtained. Our group^43^ reported an electrocatalytic strategy for upcycling ethylene glycol to glycolic acid by applying PdAg/NF as the electrocatalyst, in which the hydroxyl group is selectively oxidized while the C–C bond is maintained (R-5 in Fig. 2). PdAg/NF accelerates the ethylene glycol oxidation kinetics, which exhibits impressive ethylene glycol oxidation electrocatalytic performance with a high Faraday efficiency of 92% for glycolic acid. First, ethylene glycol undergoes dehydrogenation (C–H cleavage) due to the activity of α-H, to form intermediate 2-hydroxyacetyl species (O = *CCH_2_OH). Then OH^−^ species attack α-C of O = *CCH_2_OH through nucleophilic substitution and glycolic acid is obtained. The activity of α-H and α-C serves as a crucial determinant for the cleavage of the C–H bond and the formation of C=O/C–O bonds. Additionally, the β-H activity facilitates the formation of C=C bonds, leading to the production of olefinic compounds. The electrocatalytic oxidation of 1,3-propanediol, which has two β-H atoms, is crucial for the formation of C=C bonds. Miller^44^ developed protocols to convert 1,3-propanediol into acrylate with Pd/C–CeO_2_ as the electrocatalyst exhibiting a high selectivity of 77%. The formation of acrylate requires the elimination of both α-H, β-H combined with C–O bond cleavage to form the C=O and C=C bonds. The electrocatalytic upgrading of diols to produce high-valued products with excellent selectivity is promising.^27^

### 2.3. Electrocatalytic upgrading of polyhydroxy alcohols

In addition to mono- and diols, electrocatalysis has also been used for the upgrading of polyhydroxy alcohols. Unlike mono- and diols, the conversion of polyols is more complex and a diversity of products can be obtained. A series of valuable C_3_, C_2_, and C_1_ fine chemicals including tartronic acid (TA), lactic acid (LA), dihydroxyacetone (DHA), glyceraldehyde (GLYD), glyceric acid (GLYC), hydroxypyruvic acid (HYDP), oxalic acid (OA) and formic acid (FA) can be obtained by electrocatalytic upgrading of polyhydric alcohols, such as glycerol and sorbitol.^45–48^

#### 2.3.1. Transformation of the C–C bond

The breakage of the C–C bond is prone to occur in the oxidation of polyhydroxy alcohols. The state-of-the-art C–C bond cleavage of polycarbon molecules heavily relies on energy consumption thermocatalysis, and the selectivity of products is still unsatisfactory. Electrocatalysis offers precise control over the active site, ensuring high selectivity for C–C bond cleavage. Consequently, the efficient electrocatalytic conversion of polycarbon molecules into smaller chemical molecules holds great promise, yet it still poses significant challenges that need to be addressed. Under the catalysis of non-noble metal nickel–molybdenum–nitride nanoplates supported on carbon fiber cloth (Ni–Mo–N/CFC), C–H, C–O and stable C–C bonds of glycerol were broken successfully and value-added chemical, FA, was produced with a high Faraday efficiency of 95%.^49^ The glycerol-to-FA begins with the breakage of C–H and H–O bonds and the formation of a C=O bond, and then the obtained glyceraldehyde is oxidized to FA and glycolaldehyde. With the breakage of the C–C bond and nucleophilic substitution of α-C, glycolaldehyde is converted to FA. The electrocatalytic upgrading of sorbitol has been reported by No'e Arjona,^50^ and various products are obtained, including glycerol, ethylene glycol, formic acid, and γ-butyrolactone. In this electrocatalytic system, the Pd_*x*_Au_*y*_/C catalyst promotes C–C bond breakage, which is certainly beneficial for the production of series of value-added molecules. Cascade catalysis of electrocatalysis and acid catalysis have been exploited for C–C bond breakage. Li and co-workers^51^ exploited polyoxometalates (H_6_[PV_3_Mo_9_O_40_]) as the redox anolyte for the anodic oxidation of glucose (GOR), in which the C–C bond was broken to produce FA with a yield of 62.5%, while H_2_ was evolved continuously at the cathode. During the reaction, H_6_[PV_3_Mo_9_O_40_] plays a key role in the breakage of the C–C bond and acid catalysis is an important step for the final product generation. First, the acid catalyzes glucose to produce fructose by isomerization. Following the electrocatalysis reaction, decarboxylation of the α-hydroxy acid takes place, which subsequently leads to the breakage of the C–C bond in the α-hydroxy aldehyde. This process results in the formation of low-carbon number intermediates such as arabinose, glycolaldehyde, glyceraldehyde, and glyoxal, which are further catalytically oxidized to produce the final product FA.

#### 2.3.2. Transformation of the C–H bond

Though high value-added formate has been achieved with high selectivity by electrocatalytic upgrading of glycerol, C_3_ products with much higher value through C–H bond cleavage without C–C cleavage are also desired. With the aid of Au/Ni(OH)_2_ catalysts, Duan's group^52^ achieved a high selectivity of 77% for the conversion of glycerol to lactic acid. Glycerol is easier to form alkoxide *via* O–H bond cleavage in alkaline electrolyte. The alkoxides adsorbed on Au/Ni(OH)_2_ transfer electrons to the catalyst, resulting in the formation of a nucleophilic species, (HOCH_2_)_2_CHO*. Simultaneously, electrophilic OH* is generated and attacks the α-C of (HOCH_2_)_2_CHO* to generate DHA ((HOCH_2_)_2_CO*) along with C–H cleavage. Finally, DHA undergoes base-catalyzed dehydration and Cannizzaro rearrangement to produce lactic acid (LA) (R-6 in Fig. 2).

## 3. Electrocatalytic upgrading of carbonyls

**Fig. 3:**
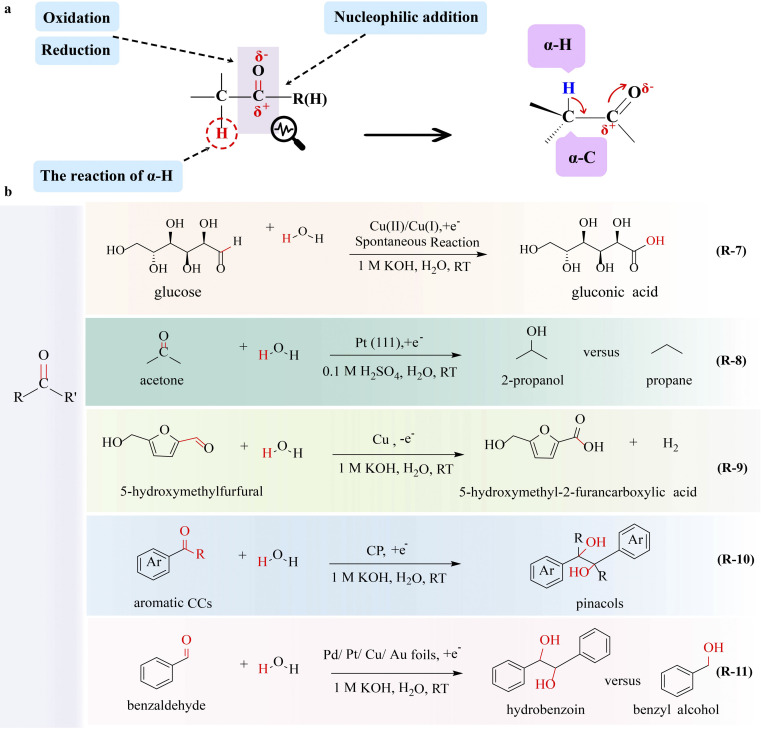
Electrocatalytic upgrading of carbonyls. (a) The illustration of chemical properties of aldehydes and ketones, and (b) the reports on carbonyl electrocatalytic upgrade.

Carbonyl is the functional group as well as the active site of aldehydes and ketones. The properties of carbonyl are very important for the conversion of aldehydes and ketones. The carbon–oxygen π bond is susceptible to electron-deficient substances, leading to reduction and addition reactions. Additionally, aldehydes and ketones undergo nucleophilic addition reactions due to higher electronegativity of oxygen compared to carbon. Conversely, carbon carries a slight positive charge, making it susceptible to nucleophilic attack. The electron-withdrawing effect of the carbonyl group activates the α-H, favoring enolization reactions in aldehydes and ketones (Fig. 3a).

### 3.1. Electrocatalytic upgrading of saturated carbonyls

Due to the presence of active α-H and electropositive carbon which is susceptible to nucleophilic attack, oxidation reactions and nucleophilic addition reactions are successfully applied for the electrocatalytic conversion of aldehydes and ketones. The oxidation of aldehydes and ketones is crucial for the synthesis of fine chemicals. In an electrocatalysis system, the electrooxidation of aldehydes and ketones usually takes place in an H-type electrolytic cell, in which the reduction of aldehydes and ketones by the cathode is hindered.

#### 3.1.1. Transformation of the C–H or/and C=O bond

Significant progress has been reported in the production of various chemicals through the electrocatalytic reaction of saturated aldehyde and ketone focusing on the C–H breakage and nucleophilic substitution at the α-C. Formate, a product of formaldehyde oxidation (FOR), is a valuable chemical. During the reaction, nucleophilic reagents tend to attack positively charged carbon, leading to the cleavage of the C=O π-bond and the formation of two σ-bonds. For two-electron-FOR electrolysis, formaldehyde undergoes partial oxidation to produce both formate and hydrogen. Sun's group^53^ reported the partial electrooxidation of formaldehyde assisted-HER system by using Cu_3_Ag_7_ as an anode electrocatalyst, which could concurrently produce formic acid and hydrogen with high Faraday efficiency. During the reaction, C–H and C=O bonds of the aldehyde group are broken to form a C–OH σ-bond with the aid of the catalyst. The stability of the electrocatalyst is very important for electrocatalytic reactions and it will be a promising strategy for utilizing *in situ* generated hydrogen at the anode to activate the electrocatalyst. Zou's group^54^ explored the conversion of formaldehyde to both formate and hydrogen at the anode. Benefiting from the *in situ* generated hydrogen at the anode, the Cu^2+^ in electrocatalyst Cu_2_O is reduced to Cu^+^, which significantly enhances the catalytic performance of Cu_2_O, exhibiting nearly 100% selectivity for formate and hydrogen production.

The electrooxidation of multivalent metals is prevalent in electrochemistry such as rechargeable ion batteries and electroplating,^55^ in which a mediator (or a redox catalyst) is used as the catalyst undergoing electron transfer to form a reactive intermediate. Then this reactive species oxidizes or reduces a substrate molecule to produce the target material homogeneously in an indirect electrolytic process with high-cost effectiveness and energy savings. A novel strategy of glucose-assisted Cu(i)/Cu(ii) redox reaction to reduce the overpotential of the GOR producing GNA has been reported,^56^ in which Cu_2_O is applied as the anode catalyst (R-7 in Fig. 3). During the electrolysis, Cu_2_O is converted to Cu(OH)_2_(Cu(ii)) by losing electrons from Cu(i), and then Cu(OH)_2_ spontaneously interacts with glucose to form Cu(i) and GNA, which allows the continuous electrooxidation of Cu(i) and completes the redox cycle of Cu(i)/Cu(ii). This system exhibits a high current density of 100 mA cm^−2^ at 0.92 V and specifically activates the aldehyde group for GNA production, without affecting the hydroxyl group.

Due to the presence of a C=O π bond, the reduction of aldehydes and ketones to alcohols and alkanes has garnered the interest of researchers. Steinberg and co-workers^57^ achieved the electrocatalytic hydrogenation of formaldehyde to methanol with a faradaic efficiency exceeding 90%, in which the C=O bond is broken to form a C–O bond. Koper and co-workers^58^ demonstrated that 2-propanol and propane can be obtained by acetone electroreduction over platinum single-crystal electrodes (R-8 in Fig. 3). The structural sensitivity of the C–C, C=O, C–O, and C–H bonds at platinum single-crystal electrodes allows for the manipulation of specific chemical bonds through the application of varying potentials. The presence of aldehyde groups in glucose makes the reduction reaction feasible. There are some research studies on the production of mannitol and sorbitol by the electrochemical reduction of glucose, in which the C=O bond is converted to a C–O/C–H bond. For example, Roesky and co-workers^59^ reported the generation of sorbitol by the electrolysis of glucose at a constant potential of 4.5 V with high conversion over 90% and yield of 80%, in which a hydrogen-storage alloy was used as a catalytic reduction electrode. The hydrogen-storage alloy electrode is highly active for the cleavage of the C=O bond affording a hydrogenation product with the C–O/C–H bond.

The conversion of FA, a promising product from CO_2_ industrial utilization, plays a crucial role in the effective utilization of carbon resources. It has been reported that the co-reduction of FA and nitrite leads to the production of high-value formamide with a selectivity of 90.0%.^60^ During the reaction, HCOO^−^ and nitrite are reduced to *CHO and *NH_2_, respectively, which couple with each other to form the key C–N coupling, leading to high-performance formamide electrosynthesis on the catalyst low-coordinated Cu.

#### 3.1.2. Transformation of the C–C bond

The electrochemical oxidation of ketone into acid by C–C bond cleavage has also been reported. Duan's group^61^ reported the oxidation of cyclohexanone to adipic acid with a high faradaic efficiency of 93% wherein a nickel hydroxide catalyst modified using sodium dodecyl sulfonate (SDS) in the interlayer (Ni(OH)_2_-SDS) served as the catalyst. During the reaction, the catalyst of Ni(OH)_2_-SDS facilitates the breakage of C–H and C–C bonds, and thus adipic acid is obtained. The oxidation of aldehydes and ketones to acids is one of the most common strategies in electrocatalytic reactions.

### 3.2. Electrocatalytic upgrading of unsaturated carbonyls

Furthermore, the electrocatalytic reactions of unsaturated aldehydes and ketones, such as cinnamaldehyde, furfural, 5-hydroxymethylfurfural (HMF) and acetophenone, are more interesting.^62,63^ Especially noteworthy is that HMF, which is the dehydration product of C_6_ carbohydrates, can serve as a versatile platform precursor for synthesizing a diverse array of commodities, including fine chemicals, plastics, pharmaceuticals, and liquid fuels.

#### 3.2.1. Transformation of the C–H bond

For instance, electrocatalytic selective oxidation of HMF (HMFOR) through C–H bond activation has been achieved to produce a series of valuable chemicals, such as 2,5-furandicarboxylic acid (FDCA), 5-hydroxymethyl-2-furancarboxylic acid (HMFCA), dialdehyde 2,5-diformylfuran (DFF), and 5-formyl-2-furancarboxylic acid (FFCA).^64,65^ It has been demonstrated that the HMFOR to FDCA is possible with a yield of 96.2% and faradaic efficiency of 93.7% by using H_2_O as the oxygen and hydrogen sources under the catalysis of CuO–PdO.^66^ The synergetic catalytic effect between CuO and PdO facilitates the breakage of C–H in the aldehyde group, forming FDCA. In addition to the aldehyde group, unsaturated aldehydes often contain other functional groups such as hydroxyl groups, unsaturated carbon–carbon double bonds, triple bonds, *etc.* According to the properties of functional groups, a specific substrate can be selectively converted to a target product at a certain potential. Wang's group^67^ adopted a simple Cu catalyst that effectively promotes the breakage of the C–H bond in the aldehyde group of HMF, leading to the concurrent production of HMFCA and hydrogen, in which the –CHO group is transformed into –COOH at relatively low potentials (R-9 in Fig. 3). By varying the potential, the aldehyde group can be selectively oxidized without affecting the hydroxyl group, allowing for the preferential conversion of HMF to HMFCA and hydrogen at the anode. To enhance the kinetics of these electrochemical reactions, high-performance catalysts are essential. Co-based catalysts have emerged as promising candidates for the HMFOR. During the electro-oxidation process, the formation of high-valence Co ions (Co^3+^ and Co^4+^) is observed, and their roles in the HMFOR are distinctly different. Deng *et al.*^65^ reported selectivity-tuned HMFOR by varying the oxidation states of CoO_*x*_H_*y*_. Co^3+^ generated at a quite low potential can act as an oxidant exclusively towards the transformation of –CHO into –COOH without oxidizing the hydroxyl group. In contrast, Co^4+^ generated at higher applied potentials plays a crucial role in initiating the oxidation of the hydroxyl group present in HMF molecules. Therefore, HMFCA and FDCA with high faradaic efficiency and selectivity can be obtained by regulating the valence state of Co.

#### 3.2.2. Transformation of the C=O bond or/and unsaturated bond

In particular, the electrocatalytic hydrogenations of HMF to 2,5-dihydroxymethylfuran (DHMF), 2,5-bis (hydroxymethyl)furan (BHMF) and 2,5-hexanedione (HD) have been verified to be feasible.^68–71^ These reactions are often accompanied by a ring–opening reaction, in which the C=O, C=C, and O–C–O bonds of the ring are broken. The conversion of HMF to HD involves the reduction of both hydroxyl and aldehyde groups, as well as the opening of the furan ring. Choi and coworkers^71^ demonstrated electrochemical conversion of HMF to HD, in which water and zinc were used as the hydrogen source and the catalytic electrode respectively. During the reaction, hydrogenolysis, Clemmensen reduction and furan ring opening (cleavage of C=C and C–O–C bonds) take place, followed by the formation of C–H and C=O bonds. The opening reaction of the furan ring involves the ring opening between C and the furan oxygen in the C–O–C bond by nucleophilic attack. The Faraday efficiency and selectivity of HD reached up to 72.4% and 81.6%, respectively, during the reduction of HMF. Holladay's group^72^ reported the electroreduction of benzaldehyde to benzyl alcohol by using a palladium electrocatalyst, in which the C=O bond is activated to form a C–OH bond. Nevertheless, innovative electrocatalytic systems are necessary to reduce energy input. Recently, a novel bifunctional Zn–organic battery has been reported by Chen group^73^ that simultaneously achieves enhanced electricity output and electrochemical reduction of a range of biomass aldehyde derivatives. With the help of a Cu NS/Cu foil electrocatalyst, the C=O bond of furfural is broken to form C–O and C–H bonds, producing furfural alcohol with a 93.5% conversion ratio and 93.1% selectivity.

Unsaturated aldehydes and ketones typically contain multiple functional groups, whose presence can influence the activity and selectivity of electrocatalytic reactions. Koper's group^74^ studied the electrochemical hydrogenation of functionalized ketones on platinum single-crystal electrodes. By defining the adsorption mode on the electrode, a new concept for electrocatalysis has been proposed that a secondary inert functional group can affect the reactivity of the other functional group and break the specific bond, and thus the desired product is obtained. The catalytic activity of an electrocatalyst crucially determines the reduction performance of unsaturated aldehydes and ketones, enabling preferential reduction of specific groups. Coincidentally, Zhang *et al.*^62^ designed CoS_2_ and CoS_2−*x*_ nanocapsules (NCs) for the electrocatalytic hydrogenation of cinnamaldehyde with excellent performance. The specific adsorption of the styryl block on pristine CoS_2_ NCs is conducive to the prioritized activation of the C=C bond, which favors the selective formation of half-hydrogenated hydrocinnamaldehyde with a 91.7% selectivity. The preferential adsorption of the C=O group, facilitated by sulfur vacancies in defective CoS_2−*x*_ NCs, results in the activation of both C=C and C=O bonds, which favors the production of fully hydrogenated hydrocinnamyl alcohol with a selectivity of 92.1%. When hydrocinnamaldehyde is the main hydrogenation product of cinnamaldehyde, flat adsorption of cinnamaldehyde is dominant on the CoS_2_ NCs; while when hydrocinnamyl alcohol is the main hydrogenation product of cinnamaldehyde, vertical adsorption of cinnamaldehyde is dominant on the CoS_2−*x*_ NCs. Furthermore, the hydrogenation method is also effective for other α,β-unsaturated aldehydes, demonstrating the versatility of the method.

The feasibility of preparing carbon-enhancing compounds through the combination of electrocatalytic reductions of unsaturated aldehydes and ketones with radical polymerization has been demonstrated. Zhang's group^75^ reported the electrocatalytic coupling reactions of benzaldehyde to pinacols (R-10 in Fig. 3). As electrocatalysts play a crucial role in enhancing selectivity, a variety of catalysts have been screened, and carbon paper has been identified to provide 99% selectivity, 96% faradaic efficiency, and a reaction rate of 0.6 mmol cm^−2^ h^−1^ towards pinacols. During the reaction, the C=O bond of benzaldehyde is activated, leading to the generation of a critical ketyl radical intermediate. The dimerization of two neighboring ketyl radicals on the CP surface results in the formation of a pinacol product (C–C coupling) that is subsequently released. The CP plays a crucial role in the selective production of pinacols; otherwise both benzyl alcohol and pinacols will be produced. To enhance the kinetics of these electrochemical reactions, further improvement of the electrocatalytic performance of the catalyst is necessary. Excitingly, Xu and co-workers^76^ proposed that the catalytic performance in producing and stabilizing ketyl radical intermediates is important for mediating the C–C coupling chemistry of aldehydes and ketones. The electrocatalytic reduction of benzaldehyde serves as a model reaction to investigate reductive C–C coupling to produce a diol product, in which the Cu catalyst stabilizes the ketyl radical, thereby facilitating the C–C coupling of benzaldehyde (R-11 in Fig. 3). By employing these radical cascade reactions, the objective of increasing the molecular weight of substrates through C–C bond formation can be achieved.

## 4. Electrocatalytic upgrading of ethers

**Fig. 4:**
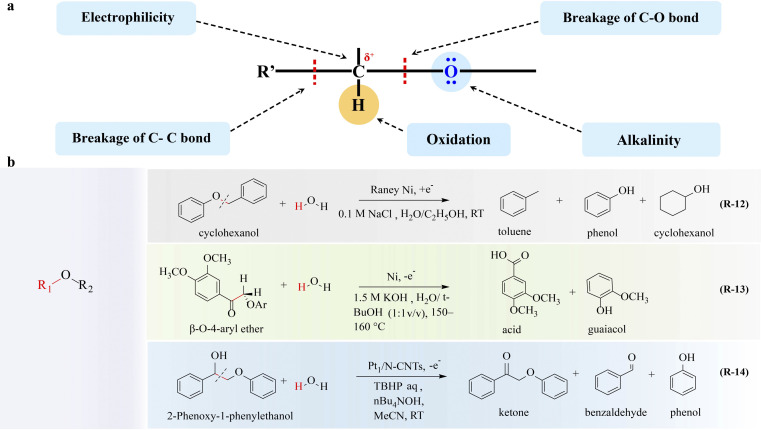
Electrocatalytic upgrading of ethers. (a) The illustration of chemical properties of ether, and (b) the reports on ether electrocatalytic upgrade.

The ether group is the functional group and the active site of the ether. As the electronegativity of oxygen is greater than that of carbon, the carbon–oxygen bond can be heterogeneously cleaved under appropriate conditions, resulting in a nucleophilic substitution reaction on the saturated carbon atom. Additionally, the α-C of ethers is susceptible to oxidation when hydrogen is present due to its electronic effect. The properties of the ether group are very important for the conversion of ethers and the electrocatalytic upgrading of ethers focusing on lignin (Fig. 4a).

### 4.1. Electrocatalytic upgrading of lignin

The lignin molecule is randomly bonded by three phenylpropane structural units, guaiacyl propane, syringyl propane, and *p*-hydroxyphenyl propane, of which the C–O ether bond accounts for about 2/3, and the rest are C–C bonds, including β-O-4, α-O-4, 4-O-5, β-β, β-5, 5-5, β-1 bonds, *etc.*^41,77,78^ The breakage of these linkages is possible depending on the different electronegativities of carbon and oxygen. By cleaving these linkages, small molecule depolymerization products containing various functional groups such as aromatic groups, methoxy groups, hydroxyl groups, carbonyl groups, and carboxyl groups can be obtained. Due to the C/H ratio of these small-molecule compounds is similar to that of petroleum, they have great application potential in the fields of high-quality liquid fuel and high-value-added chemical production. Lignin conversion is regarded as one of the most promising strategies for carbon resource upgrading. The conversion of lignin into chemical and fuel products necessitates effective methods for lignin depolymerization. Electrochemical strategies play a crucial role in enhancing lignin depolymerization and upgrading.

#### 4.1.1. Transformation of the C–O bond

The electrocatalytic method for lignin degradation and upgrading offers an attractive route for producing valuable cyclic and aromatic compounds such as phenol, toluene, cyclohexanol, and cyclohexanone.^41,79,80^ To obtain the above-mentioned products, the kinetics of aryl ether bond (C–O–C) hydrogenolysis should be faster than that of aromatic ring (C=C) hydrogenation;^81^ therefore the adsorption strength and configuration of reactants and intermediates should be optimized. Brossard^82^ reported that the electron-rich Ni electrocatalyst exhibited high efficiency toward cleavage of the C–O bond in aryl ethers (R-12 in Fig. 4). Also, the type and position of various functional groups (*e.g.*, methoxy, methyl, phenolic, and aniline groups) affecting the electronic structures of aromatic rings and O–C–O are decisive to the catalytic conversion process; thus through reasonable functional group modification, the C–O bond in the ether can be broken to obtain high value products.

#### 4.1.2. Transformation of the C–C bond

Selective cleavage of C–C linkages is a crucial step, but it remains a challenge in lignin degradation for obtaining value-added aromatic compounds. In particular, the C–C bond typically has a higher dissociation energy compared to the C–O bond in lignin, hindering the selective cleavage of the C–C bond. In this regard, various strategies, such as hydrolysis, pyrolysis, reduction, and oxidation, have been developed.^83,84^ Electrocatalytic oxidation is a potentially promising technique to cleave the C–C linkage while preserving the aromatic ring structure, transforming lignin into highly functionalized monomeric aromatic compounds.^14,85–88^ Pardini and co-workers^89^ reported electrocatalytic oxidation of a lignin β-O-4 dimeric model compound using a bulk Ni electrocatalyst. This study targeted the cleavage of the C_α_–C_β_ bond and achieved a 20.9% yield of aromatic aldehydes and carboxylic acids (R-13 in Fig. 4). A similar C_α_–C_β_ bond cleavage reaction was reported by the same group and Pt foil was explored as the anode electrocatalyst which delivered an improved yield of aromatic aldehyde.^90^ Alternatively, Rochefort and co-workers^91^ reported an indirect electrooxidation approach by using 2,2′-azinobis(3-ethylbenzthiazoline-6-sulfonate) (ABTS) as a redox mediator to facilitate C_α_–C_β_ bond cleavage with 32.5% yield of the aldehyde product.

To further elevate the conversion rate and selectivity of ether oxidation, electrocatalysts have gained great attention. Li and coworkers^92^ showed that Pt_1_/N-CNTs exhibited high activity and selectivity towards C_α_–C_β_ bond cleavage in β-O-4 model compounds (R-14 in Fig. 4). Pt_1_/N-CNTs achieves 81% yield of benzaldehyde arising from the atomically dispersed Pt–N_3_C_1_ sites, which facilitates the formation of a crucial C_β_ radical intermediate. Then this intermediate promotes a radical/radical cross-coupling pathway, ultimately leading to the cleavage of the C_α_–C_β_ bond.

## 5. Electrocatalytic upgrading of hydrocarbons

In addition to the above organics, a lot of exciting progress has also been achieved in the electrocatalytic upgrading of other kinds of compounds, especially hydrocarbons.

### 5.1. Electrocatalytic upgrading of alkenes and alkynes

**Fig. 5:**
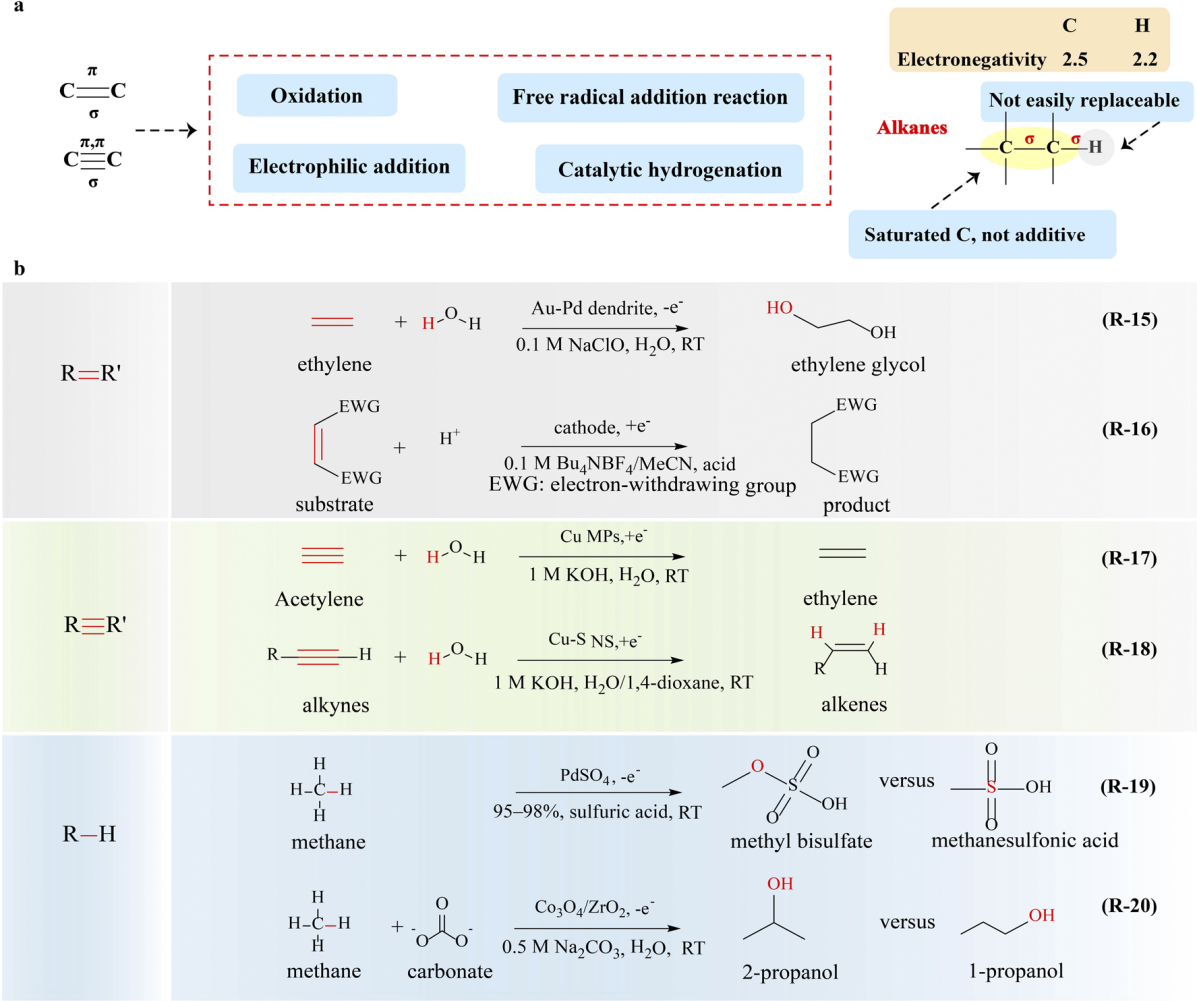
Electrocatalytic upgrading of alkenes, alkynes and alkanes. (a) The illustration of chemical properties of alkenes, alkynes, and alkanes, and (b) the reports on alkenes, alkynes and alkanes electrocatalytic upgrade.

The carbon–carbon double/triple bond is the functional group as well as the active site of alkenes and alkynes (Fig. 5a). Compared to the carbon–carbon σ bond, the dissociation energy of the π bond in alkenes and alkynes is lower, leading to a more reactive π bond. It is necessary to study the properties of the carbon–carbon double/triple bond for the upgrading of alkenes and alkynes.

#### 5.1.1. Transformation of the C=C bond

Selective oxidation of alkenes is a vital strategy for the production of aldehydes, alcohols, and epoxides, involving the breakage of C=C bonds and the formation of C–O bonds.^93^ Traditional thermal catalysis of alkenes suffers from serious environmental pollution and poor selectivity, and novel strategies are urgently needed. Recently, electrocatalytic routes using water as the sole oxygen atom source (*O or *OH) for several alkene oxidation reactions have been employed exhibiting high selectivity and yield.^94–97^ Sargent and coworkers^94^ reported one-step selective electrooxidation of ethylene to ethylene glycol in aqueous media by activating the C=C bond (R-15 in Fig. 5), in which a gold-doped palladium was used as a catalyst with a faradaic efficiency of 80%. Simultaneously, the counter electrode continuously generates hydrogen, when the selective electrooxidation of ethylene is coupled with the HER. In the selective electrooxidation of ethylene to ethylene glycol, the breakage of the C=C bond and the formation of the C–O bond are achieved through the successive transfer of two OH groups to ethylene. Apart from the design of the catalyst, the ion effect of electrolyte is also a key factor in the alternation of the reaction pathway and mechanism. The same group^98^ achieved the conversion of ethylene to ethylene oxide by utilizing Cl^−^ as the redox mediator at the anode. The generated HOCl from Cl^−^ reacts with ethylene to form ethylene chlorohydrin (HOCH_2_CH_2_Cl), which then undergoes a reaction with OH^−^ to generate ethylene oxide, and chloride returns to the Cl^−^ state for the next cycle of the reaction. This system employs chloride as the redox mediator at the anode, enabling the selective partial oxidation of ethylene to ethylene oxide with a faradaic efficiency of 70% and product specificity of 97%. The preparation of epoxides from propylene is also feasible. Epichlorohydrin, an essential industrial feedstock, has been reported by Zhang and co-workers through Br radical-mediated electrosynthesis.^99^ During the reaction, ˙Br, which activates and adds to the double bond of chloropropene, causes the formation of carbon radicals and then condenses with *OH from water oxidation to produce bromohydrin over the anode. Subsequently, hydrogen evolution on the cathode releases lots of OH^−^ ions, which makes the electrolyte alkaline. Thus, HBr will be spontaneously eliminated from bromohydrin under alkaline condition to synthesize epichlorohydrin and release Br^−^ for the next route. This strategy for the electrosynthesis of epichlorohydrin, utilizing NiCo_2_O_4_ NTs as the catalyst, demonstrates remarkable performance, achieving a faradaic efficiency of 47% and a rapid reaction rate of 1.4 mol h^−1^ g_cat_^−1^ when operated at a high current density of 100 mA cm^−2^. It exceeds the profitable target set by techno-economic analysis, thus validating its feasibility and profitability.

Due to the high reduction potentials of the C=C bond, the electrocatalytic reduction of the unactivated C=C bond is extremely difficult. Nevertheless, the C=C bond will become relatively fragile when substituted with electron-withdrawing groups, making it more susceptible to cathodic reduction.^100^ In the presence of a hydrogen donor, the hydrogenation of alkene can proceed smoothly. For example, electro-hydrogenation of activated alkenes has been reported by Tajima and co-workers,^101^ and polymer-supported sulfonic acid (Si-SO_3_H) (R-16 in Fig. 5) was used to promote the protonation step. Dimethyl maleate with two electron-withdrawing ester groups was used as the feedstock, which was converted to radical anions while the C=C bond was broken at the cathode. The added Si–SO_3_H promotes the protonation step in electroreductive hydrogenation of dimethyl maleate to minimize and even eliminate the competing coupling of the radical anion intermediate and its polymerization.^102^ Electrochemical dimerizations of trimethyl aconitate,^103^ methyl cinnamate,^104^ flavone,^105^ α,β-unsaturated ketones,^106^ and nitroolefins^107^ have also been reported by similar approaches. The electrocatalytic hydrogenation of alkenes using water as the hydrogen source shows significant advantages.

#### 5.1.2. Transformation of the C≡C bond

Selective hydrogenation of alkynes to alkenes (HEA), in which the hydrogen source attacks the C≡C bond to form the C–H bond, is a fundamental and significant transformation in synthetic chemistry.^108,109^ Traditional thermal catalysis of HEA suffers from serious pollution and poor selectivity, and thus it is of great significance to develop a more economical, energy-efficient, and highly selective route.^110^ Electrocatalytic HEA with water as the hydrogen source using a low-cost noble-metal-free catalyst is highly desirable. Deng's^111^ group investigated an efficient and selective electrocatalytic process for HEA using carbon-supported Cu microparticles (MPs) as the catalyst (R-17 in Fig. 5). During the reaction, electron transfer occurs from the Cu surface to the adsorbed acetylene, leading to preferential adsorption and hydrogenation of the acetylene over hydrogen formation and favoring the C≡C cleavage and C–H formation through the electron-coupled proton transfer mechanism. A faradaic efficiency of 83.2% for the production of ethylene has been achieved. Nevertheless, though the electrocatalytic HEA process is able to produce alkenes, the faradaic efficiency and selectivity of this system are still unsatisfactory. Zhang and co-workers^112^ succeeded in electrocatalytic semi-hydrogenation of alkynes with over 99% selectivity using water as the hydrogen source when surface sulfur-doped and adsorbed Cu nanowire sponges (Cu-S NSs) were used as catalysts (R-18 in Fig. 5). In order to evaluate the practical feasibility of this electrochemical process, the same group^7^ conducted the techno-economic analysis of electrocatalytic HEA. Feverishly, results show that this process becomes profitable if the Faraday efficiency exceeds 85% at a current density of 0.2 A cm^−2^. A Cu nanoparticle catalyst with coordinatively unsaturated sites was designed for the reaction, which shows a high C_2_H_4_ yield rate of 70.15 mmol mg^−1^ h^−1^ and a Faraday efficiency of 97.7% at an industrially relevant current density of 0.5 A cm^−2^. This electrocatalytic selective hydrogenation strategy is highly versatile and can be applied to synthesize a wide range of alkenes and alkanes with high yield.

### 5.2. Electrocatalytic upgrading of alkanes

The cleavage of C–C and C–H bonds in alkanes requires about 347 kJ mol^−1^ and 308–435 kJ mol^−1^ of bond dissociation energy, respectively, and thus alkanes have great chemical stability and do not react with strong acids, strong bases, commonly used oxidants and reducing agents. In addition, the electronegativity difference between carbon and hydrogen is negligible in alkanes, thus there is no special affinity for nucleophiles or electrophiles (Fig. 5a). On the other hand, under the action of electricity, homolytic cleavage of bonds can occur to generate free radicals inducing radical reactions.

#### 5.2.1. Transformation of the C–H bond

The electrocatalytic oxidation of alkanes is a highly studied topic, which can lead to the production of various products, such as aldehydes, ketones, alcohols, and alkenes (*e.g.*, CH_3_OH, HCHO, C_2_H_4_, C_2_H_5_OH, and C_3_H_7_OH) by electrocatalytic C–H bond breakage.^113,114^ Surendranath and coworkers^115^ introduced rapid electrocatalytic oxidation of methane to methanol precursors catalyzed by PdSO_4_ in concentrated sulfuric acid at high methane pressure (R-19 in Fig. 5). During the reaction, the conversion of PdSO_4_ into putative Pd_2_^III,III^ species rapidly activates methane, overcoming a energy barrier of 25.9 (±2.6) kcal mol^−1^ to form methanol precursors methyl bisulfate (CH_3_OSO_3_H) and methanesulfonic acid (CH_3_SO_3_H) *via* concurrent faradaic and nonfaradaic reaction pathways, in which generation of electrophilic high-valent metal species to break the C–H bond overcomes the rate-limiting redox step in thermocatalysis by electrochemical polarization featuring electron transfer. The activity of the reaction relies on the availability of *O species to react with methane, necessitating the adsorption of oxygen on the catalyst material. SnO_2_ and TiO_2_ are promising candidates for the electrocatalytic oxidation of methane, which favor C–H bond breakage.^116^

Apart from the formation of methanol and its derivatives, others products with multi-carbon atoms are also expected, such as C_2_H_4_, C_2_H_5_OH, and C_3_H_7_OH.^117^ The adsorption and conversion of the *CH_3_ intermediate are the keys to modulate the selectivity of products. Notably, during methane oxidation, certain reaction steps, particularly C–C coupling, may be homogeneous or surface mediated (*e.g.*, nucleophilic addition and free radical addition);^118^ thus the potential integration of heterogeneous electrocatalysis, homogeneous catalysis, and spontaneous reactions presents a promising avenue for enhancing the selectivity of complex chemical reactions (R-20 in Fig. 5). Although the strategy of electrocatalytic oxidation of alkanes for the production of multi-carbon products is of great significance, it still remains a challenging task and requires further exploration.

## 6. Concluding remarks

Electrocatalytic upgrading of carbon resources is an effective way for sustainable development and carbon neutrality, which necessitates great efforts from multiple disciplines. In this review, we have summarized recent advances in the electrocatalytic upgrading of carbon resources in four categories based on the types of functional groups of carbon resources, by focusing on key functional groups as the active sites of substrates and corresponding chemical bond transformations, as well as the optimization of the whole system. (i) Electrocatalytic upgrading of alcohols, (ii) electrocatalytic upgrading of carbonyls, (iii) electrocatalytic upgrading of ethers, and (iv) electrocatalytic upgrading of hydrocarbons. To enhance the efficiency of electrocatalytic upgrading of carbon resource, special attention should be paid to the following aspects:

### (1) Novel electrocatalytic upgrading configuration design

Although intensive efforts have been made to develop electrocatalytic upgrading systems and significant progress has been achieved, more novel and efficient electrocatalytic upgrading configurations should be designed for the upgrading of carbon resources. Functional groups play a decisive role in determining the properties of organic compounds, which are the core of electrocatalytic upgrading of carbon compounds. Thus, it is particularly important to make use of the specific properties of functional groups in the design of novel electrocatalytic upgrading reactions. First, thorough exploration and understanding of the properties of functional groups is the premise for the upgrading of carbon compounds. For example, aldehyde groups in aldehydes can undergo reduction at the cathode or oxidation reactions at the anode, enabling the electrocatalytic upgrading of aldehydes in a controlled manner to produce the desired product. In addition, a favorable combination of two or more transformations of functional groups in one system, as discussed above, is an efficient approach to design novel electrocatalytic upgrading configurations. For example, the co-reduction of two/more substrates at the cathode and the coupling of anodic oxidation and cathodic reduction are novel strategies for electrocatalytic upgrading, in which two or more carbon substrates are converted to the corresponding intermediates, followed by subsequent interaction of these intermediates for the target products at suitable potentials. Additionally, factors such as the electrolyte, membrane, and electrolyzer configuration should be further investigated and optimized for efficient electrocatalytic upgrading reactions.

### (2) Interaction between functional groups

Functional group conversions and interactions between different groups in the molecule(s) of substances have been considered important for electrocatalytic upgrading of carbon sources, including one molecule containing multiple functional groups, or identical/different functional groups belonging to different molecules. However, currently prevailing electrocatalytic reactions, such as aldehyde redox reactions, carbohydrate redox reactions, and aromatic compound redox reactions, focus on the conversion of single-functional groups, which suffers from limited range of reactions and/or infertile mechanisms of reactions at electrodes. Therefore, the interactions between functional groups in one electrolytic system should be brought to the forefront of electrocatalytic upgrading reactions.

### (3) Mechanism investigation

The core of electrocatalytic transformation of organic molecules is the transformation of functional groups at the catalyst–electrolyte interface featuring sequential multistep electron transfers, which mainly involve chemical bond breakage and formation. Therefore, the reaction pathways of electrocatalytic systems are closely related to the functional groups of reactants, and intensive investigations have been conducted on the reaction mechanisms and detailed reaction pathways. According to the properties of functional groups, a specific substrate can be selectively converted to desired product(s) by regulating the reaction pathway. *In situ* or *operando* observation technologies,^119^ such as *in situ* electrochemical impedance spectroscopy (EIS), Fourier transform infrared spectroscopy (FT-IR), and Raman spectroscopy, are necessary and helpful in investigating the transformation of functional groups as well as mechanisms. DFT calculations are essential tools for explaining, understanding, and predicting reaction mechanisms as well as the interactions between functional groups of substrates and intermediates.

### (4) Efficient electrocatalyst design

During electrochemical catalysis, the most important steps are the adsorption and activation of substrate molecules, which may be prevented by harsh redox potentials for the formation of intermediates. Electrocatalysts can adsorb and activate the substrates by forming a chemical bond, leading to the activation of substrate molecules at lowered overpotentials.^120^ The electrocatalytic performances of reactions can be modulated by optimizing the performances of catalysts, while the activity towards specific functional groups and the density of active sites are determined by multiple parameters such as crystal facets, electronic and surface structures, vacancy, strain, *etc.* Therefore, additional efforts should be dedicated to the development of novel and efficient electrocatalysts by investigating the structure–performance relationship.

### (5) Electrolyte

Electrolyte has a huge impact on both the yield and selectivity of electrocatalytic reactions. The local reaction environment, such as the composition and concentration of anions and cations of the electrolytes, may induce great changes in the electrostatic interactions and acidity/alkalinity. The rate of electrocatalytic reactions varies with electrolyte pH values primarily through three mechanisms.^121^ (i) Changes in concentrations of the proton donor or oxidant, (ii) adsorbate dipole–field interactions, and (iii) solution-phase reactions. The relative increments of current densities at a specific potential can vary by several orders of magnitude for several proton–electron transfer reactions on pH change (ΔpH). The acidity affects the mechanisms of electrocatalytic reactions, thus regulating the selectivity of the products.

### (6) The selection of membrane

The integration of membrane technology into electrolysis represents a groundbreaking innovation in electrochemical technology. Common categories of ion exchange membranes include the cation exchange membrane, anion exchange membrane, proton exchange membrane, and bipolar membrane. The ion exchange membrane serves to isolate two polar regions, preventing material contact between them and thus averting chemical reactions. It selectively allows ions to pass through, maintaining charge balance and establishing a closed loop. Additionally, it enables material separation and impurity removal. The selection of an appropriate ion exchange membrane plays a crucial role in determining the selectivity and conversion efficiency of the electrocatalytic reaction.

### (7) Product purification

More than one product can be generated in an electrocatalytic upgrading system, which brings about difficulties in the extraction and purification of the desired product from the mixture in most cases. Some products are highly dissoluble in the aqueous electrolytes, which should be purified or concentrated for further applications; thus a focus beyond aqueous electrolytes is crucial for purification. Solid or quasi-solid polymeric electrolytes are highly attractive for producing high-purity target chemicals, particularly when solubility is enhanced. Moreover, additional efforts are required to develop innovative and/or optimized electrocatalytic upgrading systems for the production of high-purity and/or high-concentration products in a cost-effective and practical manner.

## Author contributions

D. S. and X. T. contributed equally to this work. L. C. and J. S. structured this review. B. X. collected papers related to the topic. The manuscript was revised by all authors.

## Conflicts of interest

There are no conflicts to declare.

## Supplementary Material
